# Knockout of ABC Transporter *ABCG4* Gene Confers Resistance to Cry1 Proteins in *Ostrinia furnacalis*

**DOI:** 10.3390/toxins14010052

**Published:** 2022-01-12

**Authors:** Qing Gao, Yaling Lin, Xiuping Wang, Dapeng Jing, Zhenying Wang, Kanglai He, Shuxiong Bai, Yongjun Zhang, Tiantao Zhang

**Affiliations:** 1 State Key Laboratory for the Biology of the Plant Diseases and Insect Pests, Institute of Plant Protection, Chinese Academy of Agricultural Sciences, Beijing 100193, China; gaoqing3399@163.com (Q.G.); linyaling95@163.com (Y.L.); jingfly6@163.com (D.J.); zywang@ippcaas.cn (Z.W.); klhe@ippcaas.cn (K.H.); sxbai@ippcaas.cn (S.B.); yjzhang@ippcaas.cn (Y.Z.); 2 College of Agronomy and Biotechnology, Hebei Normal University of Science and Technology, Qinhuangdao 066000, China; 3 College of Plant Protection, Gansu Agriculture University, Lanzhou 730070, China

**Keywords:** CRISPR, *ABCG4*, *Ostrinia furnacalis*, resistant, Cry1 proteins

## Abstract

*Ostrinia furnacalis* is an important borer on maize. Long-term and large-scale planting of transgenic corn has led *O. furnacalis* evolving resistance and reducing the control effect. Recently, high levels of resistance to Bt Cry1 toxins have been reported to be genetically linked to the mutation or down-regulation of ABC transporter subfamily G gene *ABCG4* in *O. furnacalis*. In order to further determine the relationship between *ABCG4* gene and the resistance to Cry1 toxins in *O. furnacalis*, the novel CRISPR/Cas9 genome engineering system was utilized to successfully construct ABCG4-KO knockout homozygous strain. Bioassay results indicated that an ABCG4-KO strain had a higher resistance to Cry1 proteins compared with a susceptible strain (ACB-BtS). The result indicates that the *ABCG4* gene may act as a receptor of the Bt Cry1 toxin in *O. furnacalis*. Furthermore, the development time was significantly changed in the early stage ABCG4-KO larvae, and the population parameters were also significantly changed. In summary, our CRISPR/Cas9-mediated genome editing study presents evidence that *ABCG4* gene is a functional receptor for Bt Cry1 toxins, laying the foundation for further clarification of the Bt resistance mechanism.

## 1. Introduction

The Asian corn borer (ACB), *Ostrinia* *furnacalis* (Lepidoptera: Crambidae), is a worldwide agricultural pest that causes damage from East and Southeast Asia to the Western Pacific islands [1]. Larvae are the main pest on maize and seriously occurring in China [2]. Chemical insecticides are used to control *O. furnacalis*, which has led to several problems, including environmental pollution, the occurrence of resistance, and loss of biodiversity [3]. *Bacillus thuringiensis* (Bt) is a ubiquitous gram-positive bacterium, which can specifically kill target pests and is harmless to non-target insects [4]. Bt crops can not only protect the ecological environment but also increase the economic benefits [5]. Therefore, Bt bio-insecticides are a substitute for chemical insecticides for pest control. Bt produces insecticidal crystalline proteins during sporulation. The crystalline proteins are dissolved to a fragment after being ingested by an insect. The active fragments are processed by a proteolytic enzyme, binding to specific receptors on the brush boundary membrane of the midgut epithelium. The osmotic balance is destroyed, the epithelial cells are dissolved, and they damage the midgut, eventually causing larval death [6,7]. Bt toxin receptors play an important role in toxin specificity and toxicity, and mutations in these genes lead to high levels of resistance in many insect species. Bt resistance has been reported in lepidoptera and many other insects. *Plutella xylostella* was the first documented pest that developed Bt resistance in the field, repeated application of Bt protein insecticide produced significant resistance in *P. xylostella* [8,9,10,11,12].

Insects can quickly evolve resistance to Bt crops, and the efficacy of an extraordinarily specific and environmentally safe microbial insecticide may be rapidly lost to pest resistance if Bt is not deployed wisely [13,14]. A recent study indicated that ABC transporters, the largest transport family in organisms, play a central role in the pest killing mechanism. Based on the sequence similarity of nucleotide binding domains (NBD) and transmembrane domains (TMDs), the ABC transporter family includes ABCA–ABCH subfamilies [15]. Some of these have been associated with resistance to xenobiotics. In *Laodelphax striatellus*, ABCB/C/D/G genes were up-regulated in all three resistant strains, showing that ABC transporters might be involved in resistance to multiple insecticides [16]. In bed bugs, the ABC transporters were significantly up-regulated in pyrethroid resistant larvae compared with the susceptible strain. Knocking down of two ABC transporters can significantly increase the susceptibility to pyrethroid [17]. Recently, after knocking out both *ABCC2* and *ABCC3* genes by CRISPR/Cas9, can cause high levels of resistance to Cry1 toxins in *P. xylostella*, which indicates that Bt resistance was genetically linked to the mutation or down-regulation of the two genes [18]. Jin et al. [19] indicated that resistance to Cry1 toxins was genetically linked to the mutation or down-regulation of *ABCC2* and *ABCC3* genes in *Spodoptera frugiperda*. Moreover, in *Helicoverpa armigera*, simultaneous knocking out of *ABCC2* and *ABCC3* genes produced high resistance to Bt toxin Cry1Ac. However, knocking out *ABCC2* genes on their own caused low resistance, whereas knocking out *ABCC3* genes alone caused no resistance [20]. There have been fewer reports about other ABC subfamily genes involved in Cry1 toxins resistance. ABC transporters have also been shown to affect insect growth and development. In *Tribolium castaneum*, ABCG and ABCH proteins have been shown to be involved in the transport of cuticle lipids, eye pigment precursors, and ecdysides [21]. After knocking down of *TcABCG-4F* gene, *T*. *castaneum* larvae died and growth period significantly increased during adult emergence [22]. Ewart et al. [23] indicated that an ABC transporter was found to be involved in *Drosophila melanogaster* eye pigment precursor transport. Tsuji et al. [24] successfully identified one gene that plays a major role in eye pigmentation *white* orthologs in *Harmonia axyridis*.

Many genes are down-regulated in resistant strains, resulting in reduced binding to Bt proteins and resistance increased. As in the previous report, the ABCG subfamily genes were shown to be significantly down-regulated in the Cry1Ac resistant strain and may be involved in Cry1 toxins resistance [25]. For further study on the function of the *ABCG* subfamily genes in *O. furnacalis*, the CRISPR/Cas9 system was successfully used to knock out *ABCG4* gene to construct an ABCG4-KO strain. Cry1 toxins were used to test the effect of the ABCG4-KO strain compared with susceptible strains. The effects of knock out *ABCG4* gene in Asian corn borer on the growth and development were evaluated. Our result demonstrates a clear function of *ABCG4* gene in *O. furnacalis.*

## 2. Results

### 2.1. Establishment of Homozygotes ABCG4-KO Knockout Strain

**Figure 1:**
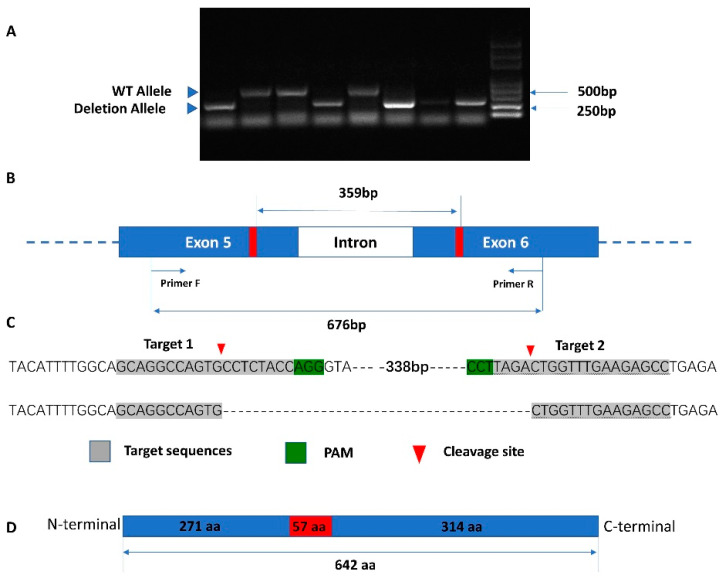
The gene knockout strategy for used CRISPR/Cas9 system in *O. furnacalis*. The gDNA (cleaved on Exon 5–6) fragments of ABCG4-KO (359 bp) were successfully detected using the gDNA extracted from leg and analyzed on 2% agarose gels (**A**). Schematic diagram exhibits the knockout fragment length by PCR amplification (**B**), sequencing detail of the cleavage site (**C**), and deletion fragment location on amino acid sequences (**D**). The deleted fragment is located on N-terminal.

After six generations (G5) of selection, a total of 120 males and females were detected by PCR and sequencing (Figure 1). In total, 80 individuals were successfully knocked out by the CRISPR/Cas9 system. The offspring of G5 were used to test the toxicity and development parameters.

### 2.2. Resistance Level of ABCG4-KO Strain to Cry1 Proteins

**Figure 2:**
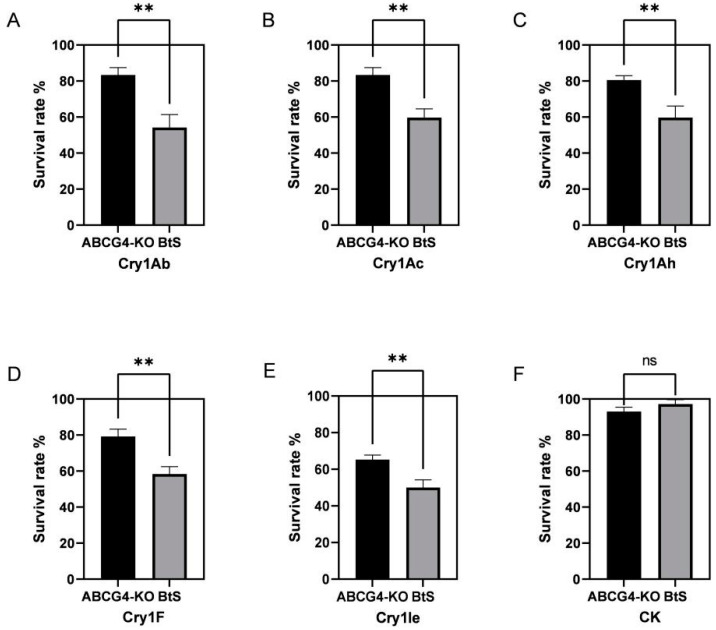
The survival rate of ABCG4-KO and ACB-BtS strains on five Cry1 proteins under LC_50_ and normal artificial diet. Survival rate of ABCG4-KO and ACB-BtS strains reared on Cry1Ab, Cry1Ac, Cry1Ah, Cry1F, and Cry1Ie protein separately (**A**–**E**). Survival rates of ABCG4-KO and ACB-BtS strains reared on normal artificial diet (**F**). ** indicates extremely significant difference between ABCG4-KO and ACB-BtS strains (*p*-value <  0.01).

The resistance level of the ABCG4-KO strain to Cry1 proteins (Cry1Ab, Cry1Ac, Cry1Ah, Cry1Ie, and Cry1F) were tested by the bioassay method. The susceptible strain ACB-BtS was used as control. Based on previous data from our lab, LC_50_ of Cry1Ab, Cry1Ac, Cry1Ah, Cry1F, and Cry1Ie virulence to ACB-BtS is 0.2 μg/g, 0.2 μg/g, 0.2 μg/g, 0.5–0.7 μg/g, and 3.0–5.0 μg/g, respectively [26]. The biometric results showed the larval survival rates of the ABCG4-KO strain on the same LC_50_ concentration as the five Cry1 proteins were significantly higher than the ACB-BtS strain (Figure 2A–E). The larval survival rates of the ABCG4-KO and ACB-BtS strains on a normal artificial diet were both above 96% and there was no significant difference (Figure 2F).

### 2.3. Larva and Pupal Development Duration and Adult Lifespan of ABCG4-KO and ACB-BtS Strain

For each of the strains, ACB-BtS and ABCG4-KO, 144 newly hatched larvae reared on artificial diet were selected. In total, 121 and 134 individuals of each strain were survived to the fifth instar, respectively (Table 1). The development time of ABCG4-KO and ACB-BtS showed significant differences in the younger (1st and 2nd) instar larvae. But there were no significant differences in other stages.

### 2.4. Pupation Rate, Emergency Rate and Oviposition Numbers per Female of ABCG4-KO and ACB-BtS Strains

The susceptible strain ACB-BtS and knockout strain ABCG4-KO successfully emergence 111 and 102 individuals, respectively. ACB-BtS and ABCG4-KO had significant differences in the pupation rate. Moreover, emergency rate and oviposition numbers per female showed no significant difference between the two strains (Table 2).

### 2.5. Population Parameters of ABCG4-KO and ACB-BtS Strains

In total, 144 individuals of the susceptible strain ACB-BtS and knockout strain ABCG4-KO were tested separately. The intrinsic growth rate, net reproductive rate, and finite increase rate were significantly decreased in the ABCG4-KO strain. However, the mean generation time was longer in the ABCG4-KO strain than in ACB-BtS (Table 3).

## 3. Discussion

The results showed that CRISPR/Cas9 editing of the *ABCG4* gene in the susceptible ACB-BtS strain significantly increased the resistance to Cry1 toxins. This information increases our understanding about the mechanism of resistance to Cry1 proteins in *O. furnacalis*. Nowadays, Bt-transgenic crops are used worldwide, and the biggest threat to Bt use is the target pests develop resistance [27]. Studying insect resistance mechanism will be helpful for improving Bt technology. Early studies showed that Alkaline phosphatase (ALP) [28], Aminopeptidase N (APN) [29], Cadherin (CAD) [30], and some other proteins are the receptors of Bt toxins [31].

Recent studies have shown that ABC transporters are one of the most important families related to Bt resistance. In *Heliothis virescens*, the mutations of the ABC transporter gene were found in resistant populations, and this mutation affects the binding of the Cry1A toxin to the brush-like membrane vesicles. This research suggests that the ABC transporter is a novel Cry1A toxin receptor that may be involved in the later stages of oligomeric membrane insertion [32]. Many ABC transporters associated with Bt resistance have been verified. The *ABCA2* gene was shown to confer resistance to Bt toxin Cry2Ab [33,34]. Zhou et al. [35] indicated that *ABCB1* is linked to resistance to Cry1Ac toxin in *P*. *xylostella*, and Jin et al. [36] indicated that *ABCB6* increases gossypol susceptibility in *H. armigera.* Zhang et al. [37] indicated that cadherin and *ABCC2* had synergistic resistance to Bt toxins in *H. armigera*. In addition, Guo et al. [38] showed that *ABCH1* was independent of Cry1Ac resistance, but the knock down of this gene could result in *P. xylostella* larval and pupal lethal phenotypes. Moreover, Shan et al. [39] showed that ABCG was related to chlorantraniliprole resistance in *P. xylostella*. Nrgri et al. [40] showed that silencing the *ABCG4* gene resulted in an increased pyrethroid efficacy. However, little research has been reported on how ABCG is related to Bt resistance. Our previous study showed that the *ABCG4* gene was significantly down-regulated in the Cry1Ac resistant strain, indicating that the *ABCG4* gene was probably related to Bt resistance [25]. In the current study, we successfully knocked out the *ABCG4* gene and constructed the ABCG4-KO strain. The survival rates of the ABCG4-KO strain after feeding on Cry1Ab, Cry1Ac, Cry1Ah, Cry1F, or Cry1Ie toxin diets were raised, and showing resistance to Cry1 proteins. This result provides evidence that the *ABCG4* gene is related to Cry1 protein resistance. The combined analysis of the down-regulated expression of the *ABCG4* gene in the resistant strain shows that *ABCG4* is probably a receptor of Cry1 proteins.

In transcriptome studies, hundreds of genes were found to be either up-regulated or down-regulated after pesticide treatment. The ABC transporter expression level changed after sublethal concentration insecticide treatments, and 80% of the significantly up-regulated ABC transporters were ABCGs [41], and in *T. castaneum*, the expression of *TcABC-C9A* was down-regulated after being treated with diflubenzuron [42]. In *P. xylostella*, the expression of four ABC transporter genes were up-regulated after treated with organophosphorus pesticide [43]. ABC transporters may be related to the transportation of xenobiotics/plant allelochemicals and insecticide resistance. Transcriptome analysis of the Cry1Ac resistant stain of *O. furnacalis* showed that *ABCG1u* gene expression was up-regulated and *ABCG4* gene expression was down-regulated [25], indicating that ABCG family genes may be related to Bt resistance.

The CRISPR/Cas9 system has been successfully used to manipulate other genes in *P*. *xylostella* and *H. armigera* to validate gene function. We used two sgRNA to knock out the *ABCG4* gene in *O. furnacalis*, and the deletion was easily detected by PCR. In *H. axyridis,* after the *ABCG-4C* gene was knocked down by RNAi, most larvae died at the pupation stage with body shrinkage [24]. Furthermore, the knocked down of *ABCG-4C* by RNA interference was fatal in *T*. *castaneum* at the pupation stage with body shrinkage. In addition, *TcABCG-4C* dsRNA-injected females laid fewer eggs and failed to hatch. The lipids were depleted in larval fat body and epicuticle after RNAi. Based on these findings, Gunnar et al. [21] suggested that *TcABCG-4C* is involved in transporting lipids to the cuticle and is essential for the formation of a waterproof barrier in the epicuticle. Moreover, Shang et al. [44] found that *ABCG4* participates in the miR-9b-ABCG4-insulin signaling pathway in aphids. Reduced *ABCG4* gene expression results in the incomplete development of aphid wings. In this study, as the *O. furnacalis* ABCG4-KO strain was still alive as the control and had no fatal effect, this indicated that *ABCG4* is not a lethal gene. However, the development times of young larvae (1st and 2nd instar) were significantly longer than the susceptible strain, and pupation rate was significantly lower than the susceptible strain. The *ABCG4* gene may be responsible for the transport of cuticle lipids, ecdysides, and other substances in the *O. furnacalis*. The result showed that the *ABCG4* gene in *O. furnacalis* may have some function in the development of young larvae. In addition, the intrinsic growth rate, net reproductive rate, and finite increase rate of the average generation time was prolonged and showed obvious resistance fitness cost. *O. furnacalis* can decrease some gene expression to obtain Bt resistance but also needs to pay the cost, for example with prolonged development or lower pupation rate. In comparing the development time in the Cry1Ac resistant strain with the susceptible stain, the resistant strain also showed a longer development time in younger larvae (data not shown). More evidence should be provided to prove whether the shortened development time is related with Bt resistance.

*O. furnacalis* is harmful to corn production and is effectively controlled by transgenic maize expression Cry1A, Cry2Ab, or Cry1F, which can effectively protect against lepidopteran pests [45]. When the use of Bt crops has increased sharply, this has been shown to be an effective way to control this pest. Our work lays the foundation for understanding that the ABC transporter *ABCG4* gene plays an important role in Cry1 toxins action mode and is a receptor gene of Cry1 toxins in *O. furnacalis*. Furthermore, ABC transporters may also be involved in insecticide susceptibility, which can be exploited for the control of *O. furnacalis*. An appropriate receptor can be used as a target gene by gene edition technology in transgene corn, which can over or suppress the target gene expression, thereby decreasing its resistance to Bt proteins and prolonging the use of Bt corn.

## 4. Materials and Methods

### 4.1. Insect Strains

*O. furnacalis* Bt toxin susceptible strain (ACB-BtS) used in the current study was fed on artificial diet for 66 generations in the laboratory of Institute of Plant Protection, Chinese Academy of Agricultural Science (CAAS). Here, we established one knockout strain (ABCG4-KO) using the CRISPR/Cas9 genome editing tool, and both strains were reared on artificial diet with a photoperiod of L/D = 16:8 h, temperature at 27 ± 1 °C, and relative humidity at 70~80%. For adults, 10% sugar solution was supplied to supplement nutrition.

### 4.2. Design and Preparation of Single Guide RNA (sgRNA)

The sgRNAs used in this study were designed following the principle of N18NGG or N20NGG protospacer adjacent motif (PAM). Two sgRNAs were used to knock out the target gene *ABCG4*; sgRNA1 and sgRNA2 were targeted at exon 5 and 6, respectively. The off target was predicted based on the similarity of genome sequences. The primers used for sgRNA synthesis are shown in Table S1. The target gene primers were mixed with DNA polymerase mix in a tube for PCR fusion. After checking the target size by 1% agarose gel electrophoresis, the PCR production was purified by DNA Purification Kit (Tianmo Biotech, Beijing, China). The purified DNA was used to synthesis the sgRNA by GeneArt^TM^ Precision gRNA Synthesis Kit (Thermo Fisher Scientific, Waltham, MA, USA) following the manufacture’s protocol. The concentration of purified sgRNA was measured by NanoDrop 2000 spectrophotometer (Thermo Fisher Scientific, Waltham, MA, USA) and stored at −80 °C until use.

### 4.3. Microinjection of Cas9 Protein and sgRNA into Embryos

Fresh egg masses (in 2 h after oviposition) were pasted on a glass slide with double-sided adhesive tape. Cas9 protein (Thermo Fisher Scientific, Waltham, MA, USA) and two sgRNA (target 1 and target 2) were mixed using the RNase-free water. Finally, the final concentration of sgRNA (200 ng/μL) was adjusted with Cas9 protein (100 ng/μL). Each embryo was injected with approximately 1 nL of the mix solution. After injection, the egg masses were incubated under 27 ± 1 °C with relative humidity at 70~80% for hatching.

### 4.4. Identification of ABCG4 Mutations Induced by CRISPR/Cas9

The microinjected eggs were placed in the incubator for hatching, the neonates were maintained in the artificial diet until pupation. After emergence, one hind leg was removed to extract DNA. Meanwhile, the adult was numbered and separately fed in a plastic box, provided with 10% honey water. Genomic DNA was extracted by M5 HiPer Mix Kit (Mei5 Biotechnology, Beijing, China) following the manufacturer’s instruction. According to the sgRNA site, primers were designed flanking the CRISPR target sites to check the deletion of *ABCG4* gene (Table S1, Supplementary Materials). The PCR reaction system (in a total volume of 20 μL) contained 10 μL of LanGene 2× Taq PCR MasterMix, 1 μL of ABCG4-F, 1 μL of ABCG4-R, 1 μL of gDNA template, and 7 μL of ddH_2_O. The PCR program was designed as follows: 94 °C for 5 min, followed by 36 cycles of (94 °C for 30 s, 56 °C for 30 s, and 72 °C for 30 s) with a final extension at 72 °C for 10 min. All the individuals were detected by PCR separately. The single PCR production band around 359 bp indicates a successfully knocked out homozygote, or around 676 bp indicates an unsuccessful knock out. Two bands indicated the sample are heterozygous.

### 4.5. Homozygous Mutant Strain Construction

To construct stable homozygous mutant strains ABCG4-KO, all the G0 adults were checked by PCR separately. The single band homozygote was sequenced and the corresponding adults were paired by one female and one male for reproducing to obtain G1 offspring. In total, 200 G1 adults were checked again and homozygotes were selected for hybridization to obtain more than 500 G2 progenies. The stable homozygous strain was selected from the progeny for large-scale hybridization until a stable homozygous mutant, ABCG4-KO, was established.

### 4.6. Bt Toxins and Bioassays

The Cry1Ab-, Cry1Ac-, Cry1Ah-, Cry1Ie-, and Cry1F-activated proteins used in all bioassays were provided by the Institute of Plant Protection, Chinese Academy of Agricultural Science (CAAS). Cry1 protein LC_50_ concentration (0.2 μg/g, 0.2 μg/g, 0.2 μg/g, 0.5–0.7 μg/g, and 3.0–5.0 μg/g of Cry1Ab, Cry1Ac, Cry1Ah, Cry1F and Cry1Ie protein, respectively) was prepared with artificial diet and ddH_2_O [26]. The control groups were fed with normal diet without any toxins. The diet used for testing was dispensed into a 24-well plate. Three plates were set for each treatment as repetition. A single newly hatched larvae were put in each well and survival rate was recorded after 7 days. Larvae mortality on control diet did not exceed 5%.

### 4.7. Life Parameter Analysis

The newly hatched ABCG4-KO strain and susceptible strain larvae were reared in a 24-well plate, with one larva per well and 144 individuals per strain. The 24-well plates were placed under 27 ± 1 °C, with relative humidity at 70~80%. The growth and development status of the two strains were recorded every day, including larval survival rate, larva stage, pupation rate, and emergency rate. After pupation, the larvae were divided into males and females and placed in a round transparent box. The emergence adults mated in a cage (80 mm × 80 mm × 100 mm) with 1:1 ratio of male and female. Oviposition paper covered the top of the cage and a wet towel was placed on it to maintain humidity. The paper was replaced every day, and the oviposition numbers per female and adult longevity were recorded. Data analysis used TWOSEX-MSChart [46,47,48] and GraphPad Prism 9.0 (GraphPad, San Diego, CA, USA). The following formula was used to calculate the life table parameters

$$\mathrm{Intrinsic}\;\mathrm{growth}\;\mathrm{rate}\;\left(r\right)=\left(lnR_{0}\right)/T$$

$$\mathrm{Net}\;\mathrm{reproductive}\;\mathrm{rate}\;\left(R_{0}\right)=\sum_{x=0}^{\infty }\sum_{j=l}^{m}S_{xj}f_{xj}$$

$$\mathrm{Mean}\;\mathrm{generation}\;\mathrm{time}\;\left(T\right)=\left(lnR_{0}\right)/r_{m}$$

$$\mathrm{Finite}\;\mathrm{increase}\;\mathrm{rate}\;\left(\lambda \right)=e^{rm}$$

## 5. Conclusions

This study provides a new *ABCG4* target gene for the sustainable utilization of transgenic plants. The *ABCG4* gene of the ACB was knocked out using the CRISPR/Cas9 technique, and the ACB showed higher resistance to five Cry1 proteins compared with a susceptible strain. The *ABCG4* gene can influence the development of younger larvae and population parameters. Our results provide a good target gene for delaying the resistance of ACB to transgenic crops, and provide a theoretical basis for the sustainable utilization of transgenic crops.

## Figures and Tables

**Table 1:** The development time of ABCG4-KO and ACB-BtS strains in larvae, pupa, and adult stages.

Different Stages	ACB-BtS		ABCG4-KO	
	*n*	Development Times (d)	*n*	Development Times (d)
1st instar	140	2.19 ± 0.03 a	131	2.67 ± 0.05 b
2nd instar	139	2.81 ± 0.03 a	130	2.34 ± 0.04 b
3rd instar	138	1.10 ± 0.03 a	129	1.17 ± 0.04 a
4th instar	137	2.00 ± 0.03 a	128	2.03 ± 0.04 a
5th instar	134	4.41 ± 0.07 a	121	5.35 ± 0.12 a
Pupa stage	130	6.71 ± 0.05 a	118	6.76 ± 0.08 a
Adult	111	6.67 ± 0.23 a	102	7.62 ± 0.27 a

Different letters in each row indicate significant difference between the two strains (analysis with *t*-test, *p*-value < 0.05).

**Table 2:** Pupation rates, emergency rates, and oviposition numbers per females of ABCG4-KO and ACB-BtS strains.

Colony	Pupation Rates (%)	Emergency Rates (%)	Oviposition Numbers per Female
ACB-BtS	90.28 ± 1.11 a	85.38 ± 2.74 a	478.98 ± 38.99 a
ABCG4-KO	81.94 ± 2.68 b	86.44 ± 2.98 a	406.00 ± 65.04 a

Different letters in each column indicate significant difference between the two strains (analysis with *t*-test, *p*-value < 0.05).

**Table 3:** Population parameters of ABCG4-KO and ACB-BtS strains.

Colony	Mean Generation Times *T* (d)	Net Reproductive Rates *R*_0_	Intrinsic Growth Rates *r*	Finite Increase Rates *λ*
ACB-BtS	26.14 ± 0.16 a	161.60 ± 24.06 a	0.19 ± 0.06 a	1.21 ± 0.01 a
ABCG4-KO	28.70 ± 0.34 b	56.53 ± 15.56 b	0.14 ± 0.01 b	1.15 ± 0.01 b

Different letters in each column indicate significant difference between the two strains (analysis with TWOSEX-MSChart, *p*-value < 0.05).

## Data Availability

All data are provided in the article and the supplementary data.

## Supplementary Materials

The following supporting information can be downloaded at: https://www.mdpi.com/article/10.3390/toxins14010052/s1, Table S1: Primers for sgRNA synthesis and detection of *ABCG4* gene deletion.
