# Supplementary material for: Knockout of ABC Transporter ABCG4 Gene Confers Resistance to Cry1 Proteins in Ostrinia furnacalis
Source: Toxins (Basel). 2022 Jan 12;14(1):52. doi: 10.3390/toxins14010052 (PMC8780026; doi:10.3390/toxins14010052)
Supplement: Supplementary file 1 [file toxins-14-00052-s001.zip › toxins-1524342-supplementary.pdf]

Supplementary Materials: Knockout of ABC transporter ABCG4 gene confers resistance to Cry1 proteins in *Ostrinia furnacalis*

Qing Gao, Yaling Lin, Xiuping Wang, Dapeng Jing, Zhenying Wang, Kanglai He, Shuxiong Bai, Yongjun Zhang and Tiantao Zhang

Table S1. Primers for sgRNA synthesis and detection of *ABCG4* gene deletion.

Target site	Primer namea	Primer sequences (5'>3')
sgRNA1	ABCG4-T1-F1	TAATACGACTCACTATAGGCAGGCCAGTGCCTC-TACC
	ABCG4-T1-R1	TTCTAGCTCTAAAACGGTAGAGGCACTGGCCTGC
sgRNA2	ABCG4-T2-F1	TAATACGACTCACTATAGGGCTCTTCAAACCAG-TCTA
	ABCG4-T2-R1	TTCTAGCTCTAAAACTAGACTGGTTTGAAGAGCC
Of ABCG4	ABCG4_104Ex5_6-F	GGTTGGATAGTTCATCGTGCTC
	ABCG4_104Ex5_6-R	AGAATCTCGTTTGGCTTTCAA

The underlined sequences were T7 promoter sequences.
